# The role of extracellular traps in ischemia reperfusion injury

**DOI:** 10.3389/fimmu.2022.1022380

**Published:** 2022-09-20

**Authors:** Feilong Zhang, Yuqing Li, Jiyue Wu, Jiandong Zhang, Peng Cao, Zejia Sun, Wei Wang

**Affiliations:** ^1^ Department of Urology, Beijing Chao-yang Hospital, Capital Medical University, Beijing, China; ^2^ Institute of Urology, Capital Medical University, Beijing, China

**Keywords:** neutrophil, NETs, extracellular traps (ETs), IRI, ischemia reperfusion injury, NETosis

## Abstract

In response to strong signals, several types of immune cells release extracellular traps (ETs), which are web-like structures consisting of DNA decorated with various protein substances. This process is most commonly observed in neutrophils. Over the past two decades, ET formation has been recognized as a unique mechanism of host defense and pathogen destruction. However, the role of ETs in sterile inflammation has only been studied extensively in recent years. Ischemia reperfusion injury (IRI) is a type of sterile inflammatory injury. Several studies have reported that ETs have an important role in IRI in various organs. In this review, we describe the release of ETs by various types of immune cells and focus on the mechanism underlying the formation of neutrophil ETs (NETs). In addition, we summarize the role of ETs in IRI in different organs and their effects on tumors. Finally, we discuss the value of ETs as a potential therapeutic target for organ IRI and present possible challenges in conducting studies on IRI-related ETs as well as future research directions and prospects.

## Introduction

Neutrophils are the most abundant immune cells in human blood. They play a central role in the innate immune defense as the first line of defense during infection and inflammation. The main modes of neutrophil function include phagocytosis, degranulation, cytokine release, and the formation of neutrophil extracellular traps (NETs) (1). NET formation is another mechanism of host defense (2). In 2004, neutrophils were first reported to kill pathogens through the formation of extracellular traps (ETs) (3). Since then, a large number of studies have focused on ETs, and it has gradually been discovered that similar to neutrophils, other immune cells (e.g., monocytes/macrophages, mast cells, eosinophils, and basophils) can also release ETs (4–7). Furthermore, researchers from various disciplines are trying to understand the ultrastructure and composition of ETs generated by various immune cells, the cellular and molecular mechanisms and signaling pathways that induce ETs, and the role of ETs in various animals and human disease states. In addition to capturing and killing microorganisms causing infectious diseases, ETs are also involved in the occurrence and development of non-infectious diseases, including autoimmune diseases (8, 9), thrombosis (10, 11), cancer (12, 13), and sterile inflammatory tissue injury (14). Among them, organ ischemia reperfusion injury (IRI) is a typical type of sterile inflammatory injury in which ETs play an important role.

Organ damage caused by ischemia/reperfusion (I/R) involves two major phases, namely, the sterile inflammatory responses due to immune cell infiltration and the oxidative stress and damage to parenchymal cells (e.g., hepatocytes and renal tubular epithelial cells) (15). Parenchymal cells and endothelial cells undergo various types of cell death under conditions of organ ischemia and hypoxia, and necrotic cells release damage-associated molecular patterns (DAMPs), including interleukin (IL)-33, heat shock proteins, histones, and high mobility group box-1 (HMGB1) (16, 17). These DAMPs promote immune cell infiltration and inflammatory factor production, further enhancing the various types of cell death of other parenchymal cells. This induces a pro-inflammatory positive feedback loop, which in turn exacerbates IRI (18, 19). Neutrophils and macrophages are the two most important types of immune cells infiltrating organs during IRI (15, 20). Several recent studies have reported that the ETs released by neutrophils and macrophages are involved in and exacerbate IRI (21, 22).

In this review, we first briefly summarize the various types of immune cells that can release ETs. Afterwards, we focus on the processes and mechanisms involved in the release of ETs from neutrophils, and their role in aggravating IRI in various organs. We further explore the link between NET formation in IRI and tumor progression. Finally, we summarize multiple approaches for the targeted inhibition of ET formation and the clearance of ET components to prevent their deleterious effects, which are of great value for mitigating IRI.

## Extracellular traps released from various immune cells

Neutrophils are the most abundant innate immune effector cells in the human immune system. They are also the most classic immune cells known to release ETs, which are web-like structures composed of DNA and granule proteins that are released after cell death (2, 3). NETs capture and kill bacteria and have an important role in the body’s intrinsic immune defense against microbial infections (2, 3). However, the excessive release of ETs by neutrophils results in blood vessel blockage, thrombosis, self-antigen exposure, parenchymal cell damage, and tumor cell metastasis, which in turn disrupt the body’s internal environment and contribute to disease development and progression (8, 12, 13, 23–25). Accumulating evidence has shown that in addition to neutrophils, other innate immune cells — including monocytes/macrophages, mast cells, eosinophils, and basophils — can also release ETs in response to various pathogenic and pro-inflammatory stimuli, which are involved in immune regulation and exert beneficial or harmful effects on the body.

Monocytes/macrophages play an important role in initiating the innate immune defense and regulating inflammation. The ability of monocytes/macrophages to release ETs has attracted increasing attention. Researchers have previously shown that statins induce the release of ETs from monocytes/macrophages by inhibiting the sterol pathway (4). Like neutrophils, macrophages can release ETs to defend the body against attack from various microorganisms, such as *Escherichia coli*, *Bacillus licheniformis*, *Haemophilus influenzae*, *Mycobacterium tuberculosis*, and *Candida albicans* (26–28). However, it has also been reported that the macrophage ETs (METs) induced by *Mycobacterium massiliense* do not have bactericidal activity and instead provide a favorable environment for bacterial aggregation and promote bacterial growth (29). Studies have also confirmed that many inflammatory mediators and chemical stimuli such as interferon-γ (IFN-γ), hypochlorous acid, IL-8, tumor necrosis factor-α, and hydrogen sulfide can also induce METs *in vitro* (30). The mechanisms of MET formation share some similarities with NETs, including NADPH/ROS-dependent mechanisms, calcium mechanisms and PAD4 mechanisms, which largely depend on the nature of the stimuli (9, 31, 32).

Mast cells are crucial for innate immune responses and are well-known for their role in initiating and maintaining local and systemic allergic responses. However, mast cells also play an equally critical role in host defense against infection, autoimmunity, and inflammatory diseases (33–35). In 2008, researchers first identified the formation of mast cell ETs (MCETs) through reactive oxygen species (ROS)-dependent cell death mechanisms. They found that the intact extracellular meshwork of MCETs can trap and effectively inhibit the growth of pyogenic bacteria (5). These MCETs are known to consist of DNA, histones, trypsin, and the antimicrobial peptide LL-37, of which LL-37 is the major effector molecule controlling Group A *Streptococcus* infection (36). Some microorganisms such as heat-killed *Mycobacterium tuberculosis* and *Listeria monocytogenes* induce the release of microbicidal MCETs by producing large amounts of ROS *via* nicotinamide adenine dinucleotide phosphate (NADPH) oxidase (NOX)-dependent mechanism (37, 38). Furthermore, the enhanced activity of the transcription factor HIF-1α induces antimicrobial effects by promoting the formation of MCETs in mice and human cells (39). During the development of psoriasis, IL-17 and IL-1β can induce the formation of MCETs *in vivo*. IL-17^+^ mast cells frequently produce IL-17 during the release of ETs, which is closely related to the pathogenesis of psoriasis (40).

Eosinophils are multifunctional cells that play an important role in the defense against parasitic infections, allergic diseases, and the protection of cardiac function after myocardial infarction and autoimmune diseases (41, 42). Eosinophils were first described to release ETs in a ROS-dependent manner in the presence of lipopolysaccharide (LPS) in combination with IL-5 or IFN-γ stimulation (6). The study showed that the release of eosinophil ETs (EETs) was independent of eosinophil death. Further, mitochondrial DNA was rapidly released from cells in a catapult-like manner, which contributed to the maintenance of intestinal barrier function and defense against bacterial infection in inflammatory conditions (6). In contrast, in human allergic diseases, local eosinophils release nuclear DNA traps after cell death (43). Studies have reported that microfilariae can trigger EETs in a Dectin-1-dependent manner, and these extracellular DNA traps can inhibit the motility of microfilariae and contribute to protective immunity against filariae (44). EETs are present in bronchoalveolar lavage fluid (BALF) derived from patients with allergic asthma, where they activate pulmonary neuroendocrine cells through the CCDC25-ILK-PKCα-CRTC1 pathway and amplify allergic immune responses (45). In tissue samples from patients with eosinophilic granulomatosis with polyangiitis, EETs with a bold net of chromatin threads are observed within small-vessel thrombi, providing a scaffold for platelet adhesion (46). In addition, EETs in diseased tissue are believed to induce elevations in cell-free DNA and the formation of immune thrombi, which is closely associated with disease activity (46).

Basophils are mainly associated with proinflammatory and immunomodulatory effects in allergic diseases and parasitic infections. Basophils, like neutrophils and eosinophils, can induce the formation of extracellular DNA traps (BETs) under the stimulation of monosodium urate (MSU) crystals (7). Recently, the physiological activation of human and mouse basophils was demonstrated to induce the release of ETs containing mitochondrial DNA and granule proteins. Moreover, BET formation was found to be independent of NOX activity (47). Furthermore, despite lacking phagocytic activity, activated basophils could kill extracellular bacteria by releasing extracellular DNA traps (48). There are relatively few studies on BETs, and substantial research is needed to explore the formation of BETs in different pathological states and their roles in the occurrence and development of diseases.

## Mechanisms underlying the formation of neutrophil extracellular traps and NETosis

The mechanism through which immune cells induce the release of ETs is not well understood. It can differ depending on the stimuli and the local microenvironment in which the different immune cells are located. In organ IRI, different infiltrating immune cells are observed at different stages following I/R. The acute phase of I/R is dominated by the inflammatory injury caused by neutrophil infiltration. A deeper understanding of the process of ET release from neutrophils and the mechanism of NET induction is essential for understanding their role and potential impact in IRI.

**Figure 1:**
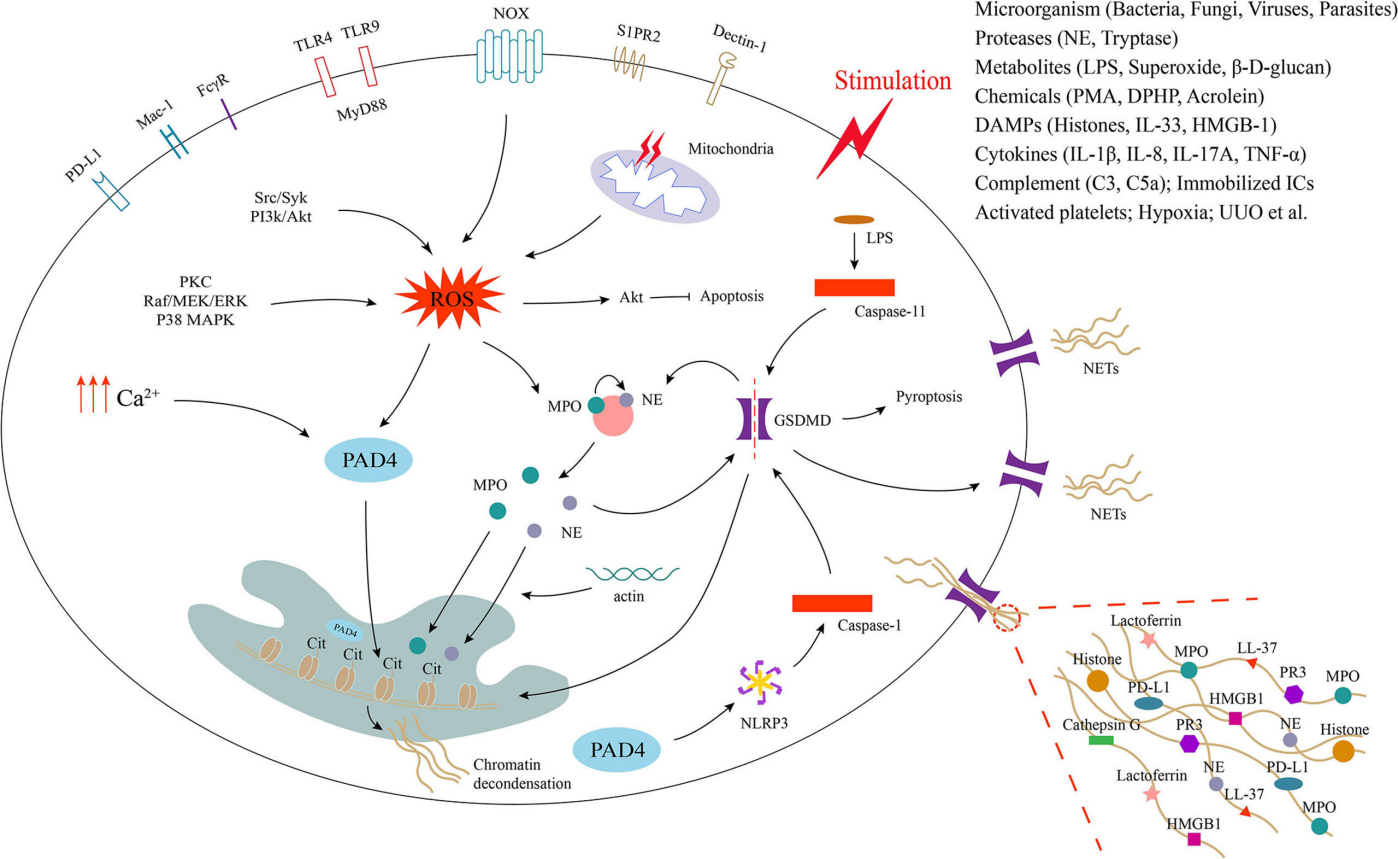
Process of neutrophil extracellular trap formation and underlying cellular and molecular mechanisms. Neutrophils release extracellular traps (NETs) by different mechanisms in response to different stimuli. Activation of neutrophil surface receptors NOX (nicotinamide adenine dinucleotide phosphate oxidase), TLRs (Toll-like receptors), FcγR (Fc gamma receptor), Mac-1 (macrophage-1 antigen), PD-L1 (programmed death ligand 1), S1PR2 and dectin-1 is involved in NET formation. The generation of ROS (reactive oxygen species) and intracellular calcium promotes the activation of PAD4 (peptidyl arginine deiminase 4), which promotes histone citrullination in the nucleus and induces chromatin decondensation. ROS production also promotes the release of MPO (myeloperoxidase) and NE (neutrophil elastase) from neutrophil granules, which enter the nucleus with the assistance of actin and also facilitate chromatin decondensation. NE and caspase-1/11 can activate GSDMD (gasdermin D), which translocated to the cell membrane to form pores, leading to membrane rupture and release of NETs decorated with various proteins such as MPO, NE, histones and more to the extracellular space.

In 2004, Brinkmann et al. (3) first reported that neutrophils release extracellular trap reticula in response to IL-8, phorbol myristate acetate (PMA), or LPS stimulation. Since then, numerous studies have reported that multiple factors can induce the formation of NETs, including various microorganisms (bacteria, fungi, viruses, and parasites), cytokines, chemicals, metabolites (lipids, cholesterol, glucose, and MSU crystals), proteases, complement, activated platelets, DAMPs, and hypoxia (19, 49–56) (**Figure 1**). The specific mechanism through which neutrophils generate NETs also differs according to the different stimuli. The release of NETs accompanied by cell membrane rupture and neutrophil death is called NETosis. Distinct from apoptosis, necroptosis, pyroptosis, and ferroptosis, NETosis is a unique cell death program observed in neutrophils. The main processes involved in NETosis are neutrophil activation, cytoplasmic granule dissolution, neutrophil protease activation, chromatin decondensation and swelling, plasma membrane rupture, and NET release (57, 58).

Studies have reported that the activation of neutrophil surface receptors such as NOX, Toll-like receptors (TLRs), Fc gamma receptor (FcγR), macrophage-1 antigen (Mac-1), programmed death ligand 1 (PD-L1), S1PR2 and dectin-1 may be involved in the initiation of NETosis, which involves the release of ROS (both NOX-derived and mitochondria-derived) or the elevation of intracellular calcium concentrations (59–62) (**Figure 1**). ROS-related upstream signaling pathways, including protein kinase C (PKC), Raf/MEK/ERK, P38 mitogen-activated protein kinase (MAPK), Src/Syk, and PI3k/Akt, may medicate the NETosis induced by PMA, immobilized immune complexes, microbes, and diphenyl phosphate (DPHP) (51, 63–66). ROS promotes the activation of Akt, which induces PMA-activated neutrophils apoptosis switch to NETosis (65). In addition, the production of ROS activates granzyme myeloperoxidase (MPO), which causes azurophilic granules to release neutrophil elastase (NE) into the cytoplasm. The activated NE is further transported to the nucleus and subsequently synergizes with MPO to promote chromatin decondensation (67–69). During this process, NE binds to and degrades F-actin to block actin dynamics (69). The inhibition of actin disassembly prevents the release of NETs (70). However, recent studies have found that NE transport to the nucleus requires the rearrangement of the actin cytoskeleton and that actin cytoskeleton dynamics are essential for NET formation (71). An increase in intracellular calcium concentrations also induces NET formation (72). Increased intracellular calcium promotes NET formation by directly activating peptidyl arginine deiminase 4 (PAD4), independent of the ROS pathway (72). Thus, PAD4 is downstream of ROS and calcium signaling during NETosis. PAD4 induces chromatin decondensation by catalyzing the conversion of arginine residues on histones to citrulline residues and is a key trigger of NETosis (73–75). Of course, NET formation can occur independent of PAD4 (66). Studies have shown that cytosolic LPS and gram-negative bacteria can drive NETosis *via* a caspase-11-dependent mechanism and the coordination of gasdermin D (GSDMD) function (76). In addition, unilateral ureteral obstruction induces NET formation *via* a caspase-11/GSDMD-dependent mechanism, which promotes renal inflammation and macrophage-to-myofibroblast transition to facilitate renal fibrosis (77). GSDMD, a pore-forming protein, can be activated upon cleavage by neutrophil proteases during NETosis and localize to the plasma membrane, causing its rupture and the release of decondensed chromatin into the extracellular space (78). The release of NETs seems to be more closely related to pyroptosis, since GSDMD is also a key regulator of pyroptosis. Furthermore, the nucleotide-binding domain (NOD)-like receptor protein 3 (NLRP3) inflammasome can also contribute to NETosis *via* a process that is dependent on PAD4, and its inhibition significantly attenuates NET formation in a noninfected state (79). Therefore, further studies are needed to better define the inflammasome pathways acting during NET formation and the possible bridging role between NETosis and pyroptosis. The mechanisms involved in NETosis are very complex, and it is likely that other synergistic or independent cellular and molecular pathways are involved in the induction of NETosis. This also needs to be further explored.

Most stimuli that induce NET formation lead to cell rupture and death, a process that takes several hours. However, as early as 2007, researchers observed that under conditions of sepsis, platelets can induce the rapid release of NETs from neutrophils within minutes through TLR4, enabling the capture of bacteria (80). Subsequently, in response to *Staphylococcus aureus* infection in human neutrophils, NETs were found to be released into the extracellular space within vesicles through a very rapid (5–60 min), unique mechanism, independent of ROS production by NOX (81). A recent study demonstrated for the first time that the rapid (within 5 min) NET release observed after exposure to *Staphylococcus aureus* is an early event in the antimicrobial response and is dependent on mitochondrial complex III (82). ROS produced by mitochondria and NOX mediate bactericidal activity in neutrophils (82). In addition, tumor-associated aged neutrophils can trigger mitochondria-dependent vital NET formation, which promotes lung metastasis in breast cancer (83). Other stimuli — including gram-positive bacteria (84), parasites (85, 86), and heparin (87) — can also induce rapid NET release. These early/rapid NET release processes have been shown to be independent of cell death, with neutrophils remaining viable for phagocytosis and chemotaxis after NET release. This type of NET release is called “vital NETosis” (23, 88). However, some experts suggest that this nomenclature is inaccurate because “osis” implies death and “vital” implies living, making these terms contradictory (89). The term “vital NET formation” is perhaps more accurate. Vital NET formation may be more closely related to infectious diseases (60). However, the rapid release of NETs has not been observed in cases of sterile inflammatory organ injury. In the future, extensive studies will be needed to clarify whether vital NET formation is present and active under conditions of normal tissue repair and organ IRI.

## Extracellular traps in organ ischemia reperfusion injury

So far, several studies have focused on elucidating the correlation between ET formation and the sterile inflammatory damage induced by organ IRI. ET formation occurs during organ I/R, aggravating organ damage, which induces the formation of more ETs. This leads to a pro-inflammatory vicious cycle and ultimately impairs organ function (14, 19). It is necessary to understand the mechanisms and signaling pathways involved in this pro-inflammatory vicious cycle formed by ETs and IRI. Therefore, we summarized the markers and mechanisms of ETs induction and inhibition in organ IRI (**Table 1**). In the following sections, we will review, in detail, the link between ET formation and IRI in various organs. Most of the literature focuses on the relationship between NETs and IRI, and this part will also be our focus of attention.

**Table 1:** Markers and mechanisms of extracellular trap induction and inhibition in organ IRI.

ETs	Organ IRI	Year	Markers in serum/supernatant	Markers in immunofluorescence	Inducers	Inhibitors	Mechanisms *in vitro*	Mechanisms *in vivo*	Refs
NETs	Liver	2015	MPO-DNA	Cit-H3 and H2AX	HMGB1, histones	NA	TLR4-/TLR9-MyD88	TLR4 and TLR9	(18)
NETs	Liver	2016	MPO-DNA	Cit-H3	Superoxide	NA	TLR4 and NOX	TLR4	(90)
NETs	Liver	2017	MPO-DNA	Cit-H3	IL-33	NA	IL-33-ST2	IL-33-ST2-MAPKs/NF-κB	(50)
NETs	Liver	2018	NA	Cit-H3, MPO and NE	Acrolein	NA	NOX2 and P38 MAPK	ERK and P38 MAPK	(91)
NETs	Liver	2018	NA	Cit-H3 and MMP9	NA	TIMP	NA	NA	(92)
NETs	Liver	2018	NET-DNA and Cit-H3	NA	Gastrointestinal MCs	NA	NA	Degranulation of gastrointestinal MCs	(93)
NETs	Liver	2019	MPO-DNA	Cit-H3	IL-17A	NA	NA	NA	(94)
NETs	Liver	2020	Cit-H3-DNA and cfDNA	Cit-H3	NA	HCQ	TLR9-PAD4/NOX	NA	(95)
NETs	Liver	2021	MPO-DNA	Cit-H3 and MPO	NA	HRG	NA	NA	(96)
NETs	Liver	2021	Extracellular DNA	Cit-H3	NA	TMP	NOX	NOX and ERK/JNK	(97)
NETs	Liver	2021	MPO-DNA	Cit-H3	NA	ExT	NA	NA	(98)
METs	Liver	2021	dsDNA, MPO and NE	Cit-H3	NA	NA	Drive of hepatocyte ferroptosis	NA	(22)
NETs	Liver	2021	Extracellular DNA and Cit-H3	Cit-H3, MPO, NE and PR3	NA	rTM	TLR4/ERK/JNK and TLR4/NADPH/ROS/PAD4	NA	(99)
NETs	Liver	2022	MPO-DNA	Cit-H3 and MPO	NA	MSC-EVs	Induction of mitochondrial fusion and enhancement mitochondrial function in neutrophils	Transfer of functional mitochondria	(100)
NETs	Kidney	2017	Extracellular DNA	Cit-H3 and NE	Platelets	NA	Activation of platelets by necrotic cell-derived DNA	NA	(101)
NETs	Kidney	2017	MPO-DNA(human), NE-DNA(mouse)	Cit-H3 and NE	Histones	NA	NA	NA	(19)
NETs	Kidney	2018	cfDNA	Cit-H3 and NE	NA	YW3-56	NA	PAD4	(21)
NETs	Kidney	2020	MPO-DNA	Cit-H3 and NE	NA	GSK484	NA	PAD4	(102)
NETs	Kidney	2020	NA	Cit-H3	NA	rTM	NA	NA	(103)
NETs	Kidney	2021	dsDNA and MPO	Cit-H3, MPO and NE	NA	Fcgr2b	Syk and NF-κB	Syk	(104)
NETs	Kidney	2021	NA	Cit-H3	P2RX1	NA	Platelets and neutrophils metabolic interaction (glycolytic metabolism and extracellular ATP)	NA	(105)
NETs	Kidney	2022	dsDNA	MPO and NE	*Candida albicans*	NA	TLR4/dectin1-Syk-NFκB	NA	(62)
NETs	Kidney	2022	NA	Cit-H3 and MPO	C3	NA	C3a-C3aR	C3a-C3aR	(106)
NETs	Intestinal	2017	MPO-Histone	NA	NA	DNase I	NA	Extracellular DNA	(107)
NETs	Intestinal	2018	cfDNA	Cit-H3	NA	DNase I	NA	NA	(108)
NETs	Intestinal	2019	NA	Cit-H3	NA	rTM	NA	NA	(109)
NETs	Intestinal	2020	NA	NA	NA	Gut microbiota	TLR4/TRIF	TLR4/TRIF	(110)
NETs	Intestinal	2020	NA	Cit-H3 and MPO	NA	TXA	ROS/MAPK	NA	(111)
NETs	Intestinal	2022	MPO-DNA and dsDNA	Cit-H3 and MPO	HMGB1	NA	NA	TLR4-MyD88	(112)
NETs	Lung	2020	CitH3-DNA and NE-DNA	Cit-H3 and NE	mtDNA	NA	TLR9 and PAD4	TLR9 and PAD4	(113)
NETs	Lung	2022	NA	NE and histones	NA	NA	NA	TLR4 and NOX4	(114)
NETs	Cerebral	2020	Extracellular DNA	Cit-H3	NA	NA	NA	PAD4	(115)
NETs	Cerebral	2022	NA	Cit3-H4 and MPO	NA	NA	NA	NA	(116)
NETs	Cerebral	2022	CitH3 and MPO-DNA	Cit-H3, MPO and NE	HMGB1	NA	NA	Platelet-neutrophil interactions	(117)
NETs	Cerebral	2022	NA	NA	PKM2	NA	STAT3 and NF-κB	NA	(118)
NETs	Cerebral	2022	NA	Cit-H3, NE and NIMP-R14	NA	NA	NA	Platelet TLR4	(119)
NETs	Myocardial	2014	NA	Cit-H3	NA	NA	NA	PAD4	(120)
NETs	Myocardial	2015	NA	DNA, histone H2B and MPO	NA	NA	NA	NA	(121)
NETs	Myocardial	2018	NA	Cit-H3	Fn-EDA	NA	NA	TLR4	(122)
NETs	Myocardial	2018	NA	Cit-H3	NA	MKEY	NA	CCL5-CXCL4	(123)
NETs	Myocardial	2020	NA	NA	NA	SPAs	NA	Histones	(124)
NETs	Myocardial	2022	MPO-DNA	Cit-H3	Gut microbiota	NA	NA	NA	(125)
NETs	Limb	2013	NA	H2A/H2B/DNA complex	NA	NA	NA	TLR4	(126)
NETs	Limb	2016	NA	H2A/H2B/DNA complex	NA	NA	NA	NA	(127)
NETs	Limb	2020	NA	Cit-H3 and MPO	NA	NA	NA	PAD4	(128)
						HCQ	NA	TLR7/8/9	
NETs	Cutaneous	2020	NA	NA	NA	mCBS	NA	Histones	(124)
NETs	Cutaneous	2022	NA	Cit-H3 and MPO	NA	IL-36Ra	NA	HMGB1	(129)

Cit-H3, citrullinated histone H3; cfDNA, cell-free DNA; C3, complement C3; DNase I, deoxyribonuclease I; ETs, extracellular traps; ERK, extracellular regulated protein kinases; ExT, exercise training; Fcgr2b, Fc gamma receptor IIb; Fn-EDA, fibronectin splicing variant containing extra domain A; HMGB1, high mobility group box 1; HCQ, hydroxychloroquine ; HRG, histidine-rich glycoprotein; IRI, ischemia reperfusion injury; IL, interleukin; IL-36Ra, interleukin-36 receptor antagonist; METs, macrophage extracellular traps; MPO, myeloperoxidase; MAPK, mitogen-activated protein kinase; MMP9, matrix metallopeptidase 9; MCs, mast cells; MSC-EVs, mesenchymal stromal cell-derived extracellular vesicles; mtDNA, mitochondrial DNA; mCBS, methyl β-cellobioside per-O-sulfate; NETs, neutrophil extracellular traps; NOX, nicotinamide adenine dinucleotide phosphate oxidase; NF-κB, nuclear factor kappa-B; NE, neutrophil elastase; NA, not available; PAD4, peptidyl arginine deiminase 4; PR3, proteinase 3; P2RX1, purinergic receptor P2X 1; PKM2, pyruvate kinase M2; Refs, references; rTM, recombinant thrombomodulin; ROS, reactive oxygen species; ST2, suppression of tumorigenicity 2; Syk, spleen tyrosine kinase; STAT3, signal transducer and activator of transcription 3; SPAs, small polyanions; TLR, Toll-like receptor; TIMP, tissue inhibitor of metalloproteinases; TMP, tetramethylpyrazine; TRIF, TIR-domain-containing adapter-inducing interferon-β; TXA, tranexamic acid.

### Extracellular traps in liver ischemia reperfusion injury

Liver IRI is a local sterile inflammatory response driven by innate immunity (15). Liver IRI usually occurs after liver resection and transplantation, and it is one of the main causes of postoperative disease recurrence and poor prognoses (130). Moreover, it is also a key contributor to early organ dysfunction and graft failure after liver transplantation (131). The mechanisms of liver IRI are complex and not fully understood. The overproduction of ROS and subsequent sterile inflammatory cascades are major contributors to tissue damage following liver IRI (132). The generation of ETs is closely related to the excessive production of ROS. ET formation is a newly discovered biological function of immune cells during sterile inflammatory responses, which is involved in the process of liver IRI.

As drivers, neutrophils play an important role in the early stages of liver I/R and are the major amplifiers of liver IRI. Neutrophils are also major contributors to the acute rejection associated with liver transplantation (133, 134). The formation of NETs plays some role in driving liver IRI (135). Excessive NET formation is often observed in the liver tissue and serum from clinical specimens and animal models of liver I/R. These NETs have been shown to be an independent factor of liver IRI (95, 100). During the initial stage of liver I/R, DAMPs (e.g., HMGB1 and histones) released by damaged hepatocytes stimulate the production of NETs through the TLR4 and TLR9-MyD88 signaling pathways (18). The resulting NETs initiate inflammatory responses and exacerbate liver injury, and PAD4 inhibitors and deoxyribonuclease I (DNase I) attenuate DAMP-mediated liver injury by inhibiting NETs (18). In a rat orthotopic liver transplantation model, HMGB1 was found to induce the formation of NETs through the TLR-4/MAPK signaling pathway (136). This promoted the intracellular translocation of HMGB1 and the M1 polarization of Kupffer cells, which in turn exacerbated acute rejection after liver transplantation (136). IL-33 is also a type of DAMP that drives neutrophil infiltration through its receptor suppression of tumorigenicity 2 (ST2) during the inflammatory response. Huang et al. (50) demonstrated that the IL-33 released by liver sinusoidal endothelial cells during liver I/R induces NET formation *via* ST2 signaling, which in turn amplifies the inflammatory cascade and sterile inflammatory response in the liver. In addition to DAMPs, other inflammatory mediators and chemicals can also exacerbate liver IRI by inducing NET formation. Using a combination of computerized dynamic network analysis and experimental validation, Tohme et al. (94) identified a central role for IL-17A in the rapid evolution of the inflammatory mediator network in the early phase of liver I/R. IL-17A exacerbates liver injury after I/R by inducing neutrophil infiltration and NET formation (94). Studies have shown that superoxide, a marker of oxidative stress after liver I/R, can induce NET formation *in vitro* through the TLR4 and NOX signaling cascade (90). In mouse models of liver I/R, pretreatment with allopurinol (superoxide inhibitor) and N-acetylcysteine (ROS inhibitor) results in a reduction of NETs and amelioration of liver injury (90). In addition, acrolein induces the release of NETs through NOX2 and P38 MAPK signaling to aggravate liver IRI in rats (91). In contrast, some physiological inhibitors and chemicals can alleviate liver IRI by inhibiting the formation of NETs. Studies have reported that tissue inhibitor of metalloproteinases-1, a physiological inhibitor of matrix metallopeptidase 9 (MMP9), can reduce the formation of NETs and thus limit the effect of NETs on the liver IRI (92). Diphenyleneiodonium (DPI), a NOX inhibitor, can inhibit the formation of NETs by inhibiting the NADPH/ROS/PAD4 signaling pathway, thereby reducing liver injury and maintaining liver function (137). Tetramethylpyrazine (TMP), the main chemical component of *Ligusticum chuanxiong*, inhibits NET formation during liver I/R by inhibiting NOX.

Further, TMP combined with DPI can effectively attenuate IRI during liver transplantation in the rat (97). Additionally, pretreatment with histidine-rich glycoproteins was found to prevent liver IRI in mice *via* the inhibition of neutrophil infiltration and NET formation (96). Hydroxychloroquine (HCQ) protects against liver IRI by blocking TLR9 to inhibit the formation of NETs (95). Recombinant human thrombomodulin prevents NET generation in neutrophils by blocking the TLR4/ERK/JNK and TLR4/NADPH/ROS/PAD4 signaling pathways, thereby preventing rat liver IRI and improving liver function (99). NETs have been shown to interact directly with platelets and exert procoagulant effects in infectious disease models. Studies have shown that NETs generated in mice with liver I/R can directly induce platelet activation *via* TLR4, leading to a systemic procoagulant state that induces remote organ injury *via* immunothrombosis (138). In a recent study, Lu et al. (100) demonstrated that human umbilical cord-derived mesenchymal stromal cell-derived extracellular vesicles exert nanotherapeutic effects, inhibiting local NET formation by transferring functional mitochondria to intrahepatic neutrophils and repairing their mitochondrial function, thereby attenuating liver IRI in mice. In addition, previous exercise training was found to reduce NET formation during liver I/R and also attenuate liver tissue necrosis (98).

The activation of liver macrophages (Kupffer cells) and resident mast cells is closely related to the accumulation of neutrophils, lymphocytes, eosinophils, and monocytes in the liver and promotes tissue injury (139). Studies have shown that the degranulation of gastrointestinal mast cells enhances the inflammatory injury cycle of liver I/R, including the hepatic infiltration of neutrophils and NET formation (93). Inhibiting the activity or number of mast cells may be an effective strategy for preventing liver IRI (93). ETs released by macrophages are also involved in liver IRI. Wu et al. (22) found that MET formation can be induced during liver I/R, leading to iron overload. This drives hepatocyte ferroptosis and ultimately aggravates liver IRI. Currently, studies on ETs and liver IRI are mainly focused on NETs. However, the liver is an immune organ and contains a large number of innate immune cells. Thus, whether mast cells, eosinophils, and basophils release ETs during liver I/R and participate in the liver injury process deserves further in-depth investigation.

### Extracellular traps in renal ischemia reperfusion injury

The formation of ETs has a broader role in the pathophysiology of several diseases involving sterile inflammation. In the kidneys, ET formation is a major driver of the self-amplifying cycle of tissue necrosis and inflammation (14). ETs are associated with many renal diseases, such as antineutrophil cytoplasmic antibody (ANCA)-associated vasculitis, immune complex glomerulonephritis, acute kidney injury (AKI) and renal fibrosis (14, 77). There exists a close relationship between renal IRI and the formation of NETs, as these events form a positive feedback loop and aggravate the renal necroinflammatory response. Studies have shown that during renal IRI, tubular epithelial cells undergo necrosis and release extracellular DNA, which causes platelet activation. The interaction of activated platelets with neutrophils causes NET formation, leading to a further increase in renal inflammation and tissue damage (101). Pretreatment with clopidogrel, which inhibits platelet aggregation prior to renal ischemia, can significantly reduce the formation of NETs in renal tissue and attenuate IRI in mice (101). Treatment with exogenous DNase I administered intraperitoneally immediately after renal I/R in rats can improve renal function and attenuate renal IRI by degrading extracellular DNA (140).

Consistent with these findings, Nakazawa et al. (19) demonstrated that the tubular epithelial cell necrosis induced during renal I/R occurs prior to the expansion of localized and circulating NETs and increased expression of inflammatory and injury-related genes. In addition, it has been revealed that extracellular histones released from dying tubular epithelial cells are central mediators in NET-related tissue damage and serve as independent acceleratory factors during the crescendo of necroinflammation in postischemic kidneys (19). Histones can induce NET formation in neutrophils, and the substances released by NETs further kill tubular epithelial cells and induce NET formation. The death of tubular epithelial cells and the production of NETs show a co-stimulatory interaction, leading to a pro-inflammatory vicious cycle that ultimately leads to renal and distal organ damage (19).

Recombinant thrombomodulin (rTM) produces anti-inflammatory effects by binding to circulating histones (141). Studies have shown that pretreatment with 10 mg/kg rTM does not ameliorate renal IRI. However, it significantly reduces the accumulation of histones and NETs in the lungs after renal I/R, exerting a protective effect on the lungs (103). PAD4 has been shown to be closely associated with NET formation in many disease models. Studies correlating renal I/R with PAD4 in mice have shown that PAD4-deficient mice do not form NETs during renal I/R, and their renal function is restored 48 h following renal I/R (21). Unlike that of PAD4-deficient mouse-derived neutrophils, the adoptive transfer of wild-type mouse-derived neutrophils to PAD4-deficient mice could restore renal NET formation and impair renal function following renal I/R (21). This cell adoptive transfer experiment confirmed that PAD4 in neutrophils plays a key role in renal IRI and NET formation (21). In addition, PAD4 is also involved in the acute lung injury (ALI) caused by renal I/R. Intraperitoneal injection of GSK484 (a PAD4 inhibitor) before renal I/R attenuates distal lung injury by reducing neutrophil infiltration, NET formation, and inflammatory cytokine secretion (102). Fc gamma receptor IIb (Fcgr2b) is associated with systemic lupus erythematosus (SLE), and Fcgr2b^-/-^ mice develop age-related lupus features. Thus, they have been used as a representative model for SLE (142). After renal I/R treatment in Fcgr2b^-/-^ lupus mice, NETs and apoptosis were found to be significantly induced in Fcgr2b^-/-^ kidneys at 24 h post-IRI, and lupus nephritis was aggravated at 120 h post-IRI (104). This process was found to be regulated by spleen tyrosine kinase (Syk) and PAD4 signaling (104). Zhuang et al. (105) systematically compared the transcriptome between IRI kidneys and sham kidneys using RNA sequencing and found that purinergic receptor P2X 1 (P2RX1) was significantly up-regulated in kidneys with IRI. P2RX1 supported the formation of NETs following renal IRI, and these NETs were essential for the impairment of mitochondrial dynamics (105). Meanwhiles, *in vitro*, the activation of P2RX1 promoted platelet ATP release, which subsequently promoted the glycolytic metabolism of neutrophils and NET formation (105). In addition, the oral administration of *Candida albicans* to mice prior to renal I/R increased systemic inflammation and NETs through the activation of TLR-4 and dectin-1, exacerbating renal IRI (62). In line with these findings, Complement C3 KO mice with renal I/R showed attenuated renal injury when neutrophil infiltration and NET formation were reduced (106).

Whether the ETs causing renal IRI are mainly derived from neutrophils or macrophages has been inconclusive. Nevertheless, most current studies have focused on the NETs promoting renal IRI. Pretreatment with anti-Ly6G IgG can deplete neutrophils in mice with renal I/R, significantly reducing renal NET production and renal injury at 24 h post-reperfusion (106). This depletion experiment demonstrated that neutrophils and their ETs play an important role in promoting renal IRI. However, in a mouse model of rhabdomyolysis, Okubo et al. (143) demonstrated that macrophages and platelets, but not neutrophils, contribute to rhabdomyolysis-induced extracellular DNA release and AKI. During rhabdomyolysis, platelet activation *via* the hemoglobin (iron) released from necrotic muscle cells enhances MET production by increasing intracellular ROS production and histone citrullination, which further promotes tubular injury (143–146). Whether macrophages and their ETs are involved in IRI independently or act in concert with NETs during renal I/R remains unclear. The numbers and proportions of neutrophils and macrophages in kidneys also vary during the different stages of I/R (20). In the early stage of renal I/R, the renal tissue is predominantly infiltrated by neutrophils, and NETs may play a dominant role during this phase. In the late stage of I/R, with the depletion of neutrophils and repair of renal tissue, the number of macrophages increases gradually. However, whether these increased macrophages can form ETs to continuously promote renal IRI or participate in tissue repair deserves further in-depth investigation. Currently, the research on NETs and METs in renal IRI is still in its infancy. A large number of studies are urgently warranted to explore the role and mechanisms of ETs in renal IRI.

### Extracellular traps in intestinal ischemia reperfusion injury

Intestinal IRI is a clinical problem that occurs most commonly after acute mesenteric ischemia, traumatic/hemorrhagic or septic shock, burns, and surgery. It can lead to multiple organ dysfunction and mortality in critically ill patients (147–149). Neutrophils may contribute to intestinal IRI by forming ETs (108, 112). After I/R induction in the rat intestine, Wang et al. (108) found that intestinal IRI leads to the excessive release of NETs. These NETs contribute to the early inflammatory response after intestinal IRI and disrupt the intestinal barrier as well as the functional integrity of tight junctions (108). The extracellular DNA released by NETs contributes to organ damage. Treatment with DNase I can disrupt generated NETs and significantly reduce the formation of NETs in the intestine and serum (108). Thus, it inhibits the histopathological changes that occur following intestinal IRI, restores the integrity of the intestinal barrier, and increases the expression of tight junction proteins (108). In addition, therapeutic interventions with DNase I attenuate tissue injury, apoptosis, and oxidative stress after intestinal I/R by inhibiting NET-mediated inflammatory responses (107). In a rat model of traumatic hemorrhagic shock, the early intravenous administration of tranexamic acid attenuated NET formation *via* the classic ROS/MAPK pathway and prevented the disruption of tight junction proteins (111). Hayase et al. (109) found that the accumulation of extracellular histones and NETs exacerbates remote liver injury after intestinal I/R. In their study, the intraperitoneal injection of 10 mg/kg rTM at the beginning of intestinal I/R in mice neutralized extracellular histones and attenuated the liver tissue injury induced by intestinal I/R (109). Using intravital imaging technology, Ascher et al. (110) found that the presence of some gut microbes restricted NET formation in I/R-injured mesenteric venules, likely due to diminished neutrophil TLR4 signaling. Furthermore, they also demonstrated that the TLR4/TRIF signaling axis was critically involved in mesenteric IRI-induced NETosis (110). Zhan et al. (112) found that NETosis was enhanced in the lungs after intestinal I/R in C57BL/6J mice and that the deletion of MyD88 attenuated the production of NETs and intestinal I/R-induced lung injury. Treatment with DNase I or a PAD4 inhibitor significantly attenuated intestinal I/R-induced ALI (112). In addition, the HMGB1 released from necroptotic enterocytes during intestinal I/R exacerbated the intestinal I/R-induced ALI by inducing NET formation (112). Therefore, NETs could serve as clinical indicators and therapeutic targets for intestinal IRI. Targeting NETosis and its products could help in attenuating intestinal I/R-induced remote organ injury.

### Extracellular traps in lung ischemia reperfusion injury

Lung IRI is a common pathological condition, and the resulting inflammatory cascade is thought to play a central role in its pathophysiology (150). Lung IRI usually occurs after lung transplantation and is one of the main factors leading to primary graft dysfunction (PGD) in recipients and early morbidity and mortality after lung transplantation (151, 152). In BALF from human lung transplant recipients, NETs were found to be more abundant among patients with PGD (153). NET formation was increased following either hilar clamp or orthotopic lung transplantation after prolonged cold ischemia (OLT-PCI) (153). Disruption of NETs *via* the inhibition of platelets or the intrabronchial administration of DNase I reduced lung injury and improved oxygenation (153). In addition, increased mitochondrial DNA (mtDNA, an endogenous DAMP) levels were detected in the BALF of an experimental PGD model induced by OLT-PCI, and it was confirmed that the mtDNA released during lung I/R triggers NET formation *via* TLR9 signaling, driving lung injury (113). TLR9 deficiency in lung recipients or donors reduces NET formation and lung injury (113). Thus, DNase I treatment may have the dual benefit of both degrading pathogenic NETs and neutralizing NET triggers such as mtDNA. Using intravital imaging, oxidative lipidomics, and transplant models, Li et al. (114) demonstrated that TLR4 signaling and downstream NOX4 expression in vascular endothelial cells during lung I/R mediate neutrophil recruitment to the lungs and increase NET formation. The knockdown of TLR4 expression in vascular endothelial cells results in decreased neutrophil infiltration and NETosis (114). Treatment with DNase I reduces lung neutrophil extravasation and subpleural NET formation, thus improving graft function (114). However, studies also show that although DNase I treatment can rapidly degrade NETs within the graft, the ensuing release of NET fragments promotes the production of inflammatory factors in human alveolar macrophages by activating the TLR-MyD88 signaling pathway (154). It also initiates the proliferative response of dendritic cells to alloantigen-specific CD4^+^ T cells, preventing lung transplant acceptance (154). In addition, Antunes et al. (155) demonstrated — for the first time — that methoxyeugenol protects lung tissue from inflammation and inhibits LPS-induced neutrophil infiltration and NET formation in ALI mice. At present, the specific mechanism underlying the induction of NETs during lung I/R and the impact of NETs on lung injury or lung transplant rejection remain unclear and need to be explored in depth. In addition, whether alveolar macrophages can also release ETs during I/R and the role they play in lung IRI deserves further investigation.

### Extracellular traps in cerebral ischemia reperfusion injury

Cerebral IRI, which usually occurs after thrombolysis and recanalization in ischemic stroke, is characterized by massive cell death and neutrophil activation. Novotny et al. (156) detected NETs within the thrombi of 100% (71/71) of patients with acute ischemic stroke (AIS) and confirmed that the abundance of NETs in these thrombi was associated with poor outcomes in these patients. In addition, the level of NETs in the thrombi was also related to the degree of neurological injury (116). With the prolongation of reperfusion, collateral blood flow improved in patients with ischemic stroke, and this was associated with lower levels of NETs in the thrombus (116). These results suggest that targeting NETs in thrombi may enable early neurological protection in AIS patients. In line with this, Denorme et al. (117) found that elevated plasma biomarkers of NETs are associated with worsening stroke outcomes. During AIS, NETs can exert deleterious effects in a platelet TLR4-dependent manner, and the early administration of DNase I can reduce infarct size and improve stroke outcomes after ligature-induced permanent middle cerebral artery occlusion (119). In addition, other studies have shown that platelets can exacerbate cerebral IRI by driving HMGB1 release and NET formation (117). Neonatal NET-inhibitory factor (nNIF) is an endogenous NET inhibitory peptide that blocks the formation of NETs without affecting the other functions of neutrophils (157, 158). Prophylactic therapy with nNIF effectively prevents platelet-induced NET formation and improves short-term and long-term outcomes following ischemic stroke (117). Polymorphonuclear granulocytes (PMNs) migrate into the brain parenchyma and release large amounts of proteases, which are thought to be the main cause of neuronal cell death and reperfusion injury following ischemia (159). However, Enzmann et al. (160) found no PMN infiltration in 25 infarcted brain tissue samples collected from patients with ischemic stroke at early post-infarction time points. Moreover, they found that intravascular PMN aggregation did not correlate spatially with the release of NETs (160). In contrast, studies have shown that neutrophils accumulate around the meninges and blood vessels after cerebral I/R and eventually reach the infarcted brain parenchyma (161). Disruption of the basement membrane and NET formation can be detected 24–48 h after reperfusion (161). In addition, the release of NETs impairs the blood–brain barrier and vascular remodeling during stroke recovery. However, the disruption of NETs using DNase I or the knockdown of PAD4 increases neovascularization and repair and improves functional recovery (115). The components of NETs, i.e., histones and extracellular DNA, are also detrimental during cerebral I/R, and targeting them can attenuate the damage caused by ischemic stroke (162). Studies have also shown that the formation of NETs after cerebral I/R is closely related to the *pyruvate kinase M2 (PKM2)* gene in myeloid cells, which regulates the post-ischemic inflammatory response of peripheral neutrophils by promoting the phosphorylation of signal transducer and activator of transcription 3 (STAT3) (118). Myeloid cell-specific PKM2^-/-^ mice show reduced formation of NETs and improved cerebral blood flow, and also exhibit reduced thrombotic inflammation following cerebral I/R (118). ML265 is a small molecule that inhibits PKM2 nuclear translocation by inducing its tetramerization (163). ML265 treatment significantly reduces the nuclear translocation of PKM2 and inhibits NETosis after AIS. Additionally, it improves long-term sensorimotor outcomes in mice (118). Currently, DNase I has been identified as the main degrader of NETs following cerebral I/R (164). Studies have shown that differently polarized macrophage subsets can degrade NETs (165). Microglia, a type of macrophage in cerebral, can protect neurons by direct engulfment of invading neutrophil (166). It would be interesting to explore whether reactive microglia can regulate the formation of NETs in cerebral IRI. The link between microglia and NETs in cerebral IRI deserves further in-depth examination.

### Extracellular traps in myocardial ischemia reperfusion injury

Innate immune cells play an important role in the early response to myocardial IRI. During myocardial I/R in mice, high neutrophil infiltration and NET formation can be observed in the injured myocardial tissue (120). The intraperitoneal injection of recombinant human DNase I at 1 h after the induction of a left anterior descending occlusion and 11 h after reperfusion can reduce the infiltration of neutrophils and the formation of NETs in myocardial tissue (120). Additionally, it can reduce the size of the myocardial infarct and improve cardiac function (120). Further, PAD4^-/-^ mice do not produce NETs during I/R and are protected from myocardial IRI (120). Previous studies have demonstrated the potential role of NETs in linking sterile inflammation to thrombosis (167). It was shown that in rats, neutrophil (MPO-positive) density in the left ventricular ischemic zone increases following 45 min of myocardial ischemia and 3 h of reperfusion, and this is accompanied by strong immunostaining for NETs (121). The intravenous administration of DNase I 5 min before reperfusion reduces I/R-induced neutrophil aggregation, NET formation, and MPO activity (121). In addition, NET-mediated microthrombosis contributes to myocardial “no-reflow.” However, DNase I combined with recombinant tissue-type plasminogen activator (rt-PA) reduces myocardial I/R-induced anatomic “no-reflow” and limits infarct size, improving long-term post-infarction left ventricular remodeling (121). Surprisingly, rt-PA treatment alone has no significant effect on the number of NETs (121). Extracellular histones in NETs are highly toxic to tissues (168). Meara et al. (124) described a non-toxic small polyanion (SPA) that interacts electrostatically with histones to displace them from NETs, thereby destabilizing their structure and neutralizing their pathological effects (124). SPAs were found to significantly inhibit rat myocardial IRI *in vivo* by reducing NET formation and free histone-mediated pathological damage (124). Fibronectin splicing variant containing extra domain A (Fn-EDA), an endogenous ligand of the innate immune receptor TLR4, can promote thrombosis and inflammation (169, 170). In one study, hyperlipidemic apolipoprotein E-deficient mice with Fn-EDA knockout showed less neutrophil infiltration and NET formation compared to WT mice after myocardial I/R. Moreover, they showed reduced cardiomyocyte apoptosis and infarct size. The findings confirmed that Fn-EDA-mediated myocardial IRI requires the involvement of TLR4 (122). The heterodimerization of platelet-derived CCL5 and CXCL4 enhances their ability to activate and recruit inflammatory cells and is involved in the formation of NETs (171). Researchers specifically designed a compound called MKEY (a peptide antagonist) to block the interaction between CCL5 and CXCL4 (172). MKEY was administered intravenously to mice 1 day before myocardial I/R and treatment was continued until 7 days after I/R. The results showed that MKEY treatment significantly reduces the inflammatory response after I/R and the formation of NETs *in vivo*, while also reducing myocardial infarct size and improving cardiac function (123). The gut microbiota plays a crucial role in cardiovascular disease. During myocardial I/R, the gut microbiota induces the formation of NETs, which can directly lead to the apoptosis of cardiomyocytes and myocardial microvascular endothelial cells, exacerbating myocardial IRI (125). In addition, Tang et al. recently showed that Kruppel-like factor 2 (KLF2)-deficient neutrophils exhibit enhanced NET formation *in vitro* and are essential for angiotensin II-induced cardiac hypertrophy (173, 174). However, whether KLF2 is involved in myocardial IRI, and whether KLF2 has a negative effect on NET formation during myocardial I/R, warrants further investigation.

### Extracellular traps in limb ischemia reperfusion injury

Limb IRI is an important clinical challenge in patients with acute muscle ischemia after trauma or a major artery thrombotic occlusion due to lower extremity vascular disease. Limb IRI can evolve to inflammation, tissue edema, muscle fibrosis, and necrosis, eventually resulting in reduced range of motion or complete loss of function (175). In a mouse model of acute hind limb I/R, IRI caused extensive immune infiltration and strong NET formation in skeletal muscle (128). Oklu et al. (126) found that WT mice experiencing limb I/R showed higher levels of NET formation than TLR4 mutant mice, and that these NETs were mainly present in the interstitial tissue and perivascular and microvascular thrombi. NETs in skeletal muscle sections from WT mice were significantly reduced after treatment with DNase I (126). These results suggest that NETs may contribute to limb I/R-induced muscle fiber injury in a TLR4-dependent manner. HCQ, a small molecule inhibitor of TLR7/8/9, has been found to reduce the formation of NETs and the production of inflammatory molecules after limb IRI, ultimately attenuating muscle fibrosis and improving muscle fiber regeneration after IRI (128). Albadawi et al. (127) evaluated the effect of exogenous DNase I treatment on skeletal muscle injury after acute limb IRI in mice. They found that although treatment with DNase I significantly reduced ET formation in ischemic muscles, it did not alter skeletal muscle fiber injury or the levels of proinflammatory molecules (127). In addition, neutrophil depletion followed by limb I/R marginally reduced ET formation in ischemic muscle, but did not alter skeletal muscle fiber injury (127). These data indicate that neutrophils are not the only contributors to ET formation and muscle fiber injury after limb IRI. A large number of studies are needed to explore the role and mechanisms underlying the induction of limb IRI *via* ETs released from other immune cells.

### Extracellular traps in cutaneous ischemia reperfusion injury

Cutaneous IRI typically occurs due to pressure ulcers, Raynaud’s phenomenon-induced skin ulcers, and skin flap grafts following reconstructive surgery. It is related to the oxidative damage caused by apoptosis, necroptosis, ROS, and excessive generation of pro-inflammatory factors (176–178). NETs have been reported to delay the healing of skin lesions, and pharmacological targeting of NETs can accelerate wound regeneration (179, 180). Methyl β-cellobioside per-O-sulfate (mCBS), a type of SPA, can inhibit NET-associated histone-mediated injury. Further, mCBS injection 5 min before and after cutaneous I/R can consistently and significantly increase the area of skin flap survival (124). IL-36 receptor antagonist (IL-36Ra), encoded by *Il36rn*, attenuates myocardial IRI though reducing neutrophil recruitment and improving blood flow in mice (181). Tanaka et al. (129) found that in Il36rn^-/-^ mice, cutaneous I/R resulted in a significant delay in wound healing and increased inflammatory cell infiltration. Furthermore, they found that compared with WT mice, Il36rn^-/-^ mice showed significantly greater NET formation in the cutaneous tissue around the IRI after 4 and 72 h of reperfusion (129). The intraperitoneal injection of Cl-amidine (10 mg/kg/day) to inhibit NET formation significantly attenuated the cutaneous IRI in Il36rn^-/-^ mice (129). These results show, to some extent, that NETs are associated with the exacerbation of cutaneous IRI. Skin flap tissue IRI caused by skin flap transplantation is one of the primary reasons for the low success rate of the procedure (176). Hence, there is a need to explore the intrinsic links between NETs and skin flap transplantation-induced IRI along with the related mechanisms. NET targeting could become a new intervention for improving skin flap survival. Furthermore, whether Langerhans cells in the skin can also release ETs and participate in cutaneous IRI is unknown and must be explored in depth.

## Link between ischemia reperfusion injury-induced neutrophil extracellular traps and cancer

Back in the mid-19th century, Rudolf Virchow discovered leukocyte infiltration in tumor tissue and first proposed the link between inflammation and cancer (182). Today, the close relationship of inflammation with the tumorigenesis and metastasis of most types of cancers has been clarified (183). IRI is a type of sterile inflammatory injury, and I/R-induced sterile inflammation promotes tumor recurrence and metastasis after liver resection or liver transplantation (184, 185). Under inflammatory conditions, the web-like DNA strands released by NETs, which are embedded with various proinflammatory molecules, capture circulating tumor cells (CTCs) and contribute to cancer metastasis (186–188). Yang et al. (189) proposed that NET-DNA is a chemokine that activates the ILK-β-parvin pathway to enhance cell motility and promote cancer metastasis by binding to the transmembrane protein CCDC25 on cancer cells. In this review, we have previously mentioned that a large number of NETs are induced during I/R. Therefore, it would be interesting to understand whether IRI-triggered cancer recurrence and metastasis are closely related to NET formation during I/R. Tohme et al. (190) observed that in patients undergoing hepatectomy for colorectal liver metastases, greater evidence of postoperative NET formation in the serum was associated with a higher risk of recurrence. Subsequently, they induced liver I/R in a mouse model of colorectal liver metastases and found that NETs formed due to IRI promoted the development and progression of liver metastases. Interestingly, this effect could be reversed through local treatment with DNase I or the inhibition of PAD4, which hinder NET formation (190). Ren et al. (191) studied the relationship between IRI-induced NETs and cancer metastasis from the perspective of platelet–neutrophil interactions. Their findings suggested that the surgical stress induced by liver I/R in mice activates platelets and promotes their aggregation within tumor cells *via* the TLR4-ERK5 axis, which is conducive for the capture of tumor cells by IRI-induced NETs and subsequent distant metastasis (191). Blocking platelet activation or the knockdown of TLR4 protects mice from liver I/R-induced metastasis, and no CTCs are captured by NETs (191). This result suggests that the targeted disruption of the interaction between platelets and NETs may have therapeutic effects, preventing postoperative distant metastases. The relevance of NETs to T cells has been studied to a limited extent. Kaltenmeier et al. (192) induced liver I/R to generate a NET-rich tumor microenvironment (TME) in an established cancer metastasis model. They found that IRI-induced NETs promote tumor growth by enhancing CD4^+^ and CD8^+^ T cell exhaustion and dysfunction in the TME, which is closely associated with the PD-L1 embedded within the NET chromatin. Targeting NETs containing PD-L1 *via* DNase or anti-PD-L1 treatment attenuates tumor growth (192). In addition, liver IRI-induced NETs also contain HMGB1, and NET-derived HMGB1 enhances tumor invasiveness by inducing the epithelial to mesenchymal transition (EMT) program (193). Thrombomodulin prevents NET-induced EMT by blocking HMGB1, thereby inhibiting the promotive effect of IRI-induced NETs on liver metastasis (193). Tohme et al. (194) found that drag reducing polymers, which are blood-soluble macromolecules, reduce IRI-induced platelet aggregation, neutrophil infiltration, and NET formation. Moreover, they also prevent I/R-induced metastatic tumor development and growth. In a metastatic model, exercise training pretreatment was found to attenuate the inflammatory response of liver IRI and the formation of NETs, reducing the incidence of IRI-related liver metastases (98). In conclusion, I/R-induced NET formation contributes to tumor development and metastasis. The alleviation of I/R-induced sterile inflammation and targeted interventions against NETs at the site of injury are emerging as promising therapeutic strategies for reducing postoperative tumor progression and recurrence. Currently, studies in this area mainly focus on liver IRI. However, whether NETs induced due to IRI in other organs also contribute to tumor development and metastasis deserves further investigation.

## Targeting extracellular traps for ischemia reperfusion injury treatment

As mentioned above, ETs are involved in the IRI process in various organs, and the excessive production and/or impaired clearance of ETs can exacerbate IRI. Therefore, strategies for reducing excessive ET formation or enhancing ET degradation are expected to improve organ IRI and have obvious therapeutic benefits. Therapeutic approaches for inhibiting the overproduction of NETs by targeting key molecules with various drugs or gene knockout technology have been reported in the literature (**Table 2**). Among the many approaches used to target intracellular signaling molecules, the drugs rTM and HCQ inhibit NET formation by inhibiting the TLR pathway (95, 99). The inhibition of genes such as IL-33, ST2, IL-36R, P2RX1, PKM2, and complement C3 *via* gene knockout technology or drugs can reduce NET formation and attenuate IRI (50, 105, 106, 118, 129). In addition, the interaction of platelets and mast cells with neutrophils can promote the formation of NETs. Thus, the inhibition of platelet activation or the elimination of platelets and mast cells can inhibit NET formation and control IRI and IRI-induced tumor metastasis (93, 101, 191). However, these interventions do not directly target NETs.

**Table 2:** Targeted anti-extracellular trap interventions to improve outcomes of organ IRI.

Target molecule/function	Agents/interventions	Effects	Organ IRI	Refs
***NET components***				
DNA	DNase I	Degradation of NETs	Liver	(18) (94, 100) (138) (190) (192)
			Kidney	(101) (140) (21) (105)
			Intestinal	(107, 108, 111, 112)
			Lung	(113) (114)
			Cerebral	(117) (119) (115) (162)
			Myocardial	(120, 121)
			Limb	(126, 127)
Histones	Anti-histones antibody: BWA3 and histones neutralizer: mCBS, rTM	Inhibition of NETs, decreased histones and NETs accumulation	Kidney	(19, 103)
			Intestinal	(109)
			Cerebral	(162)
			Myocardial	(124)
			Cutaneous	
Histone citrullination	Pan-PAD inhibitors: Cl-amidine, YW3-56, YW4-03	Inhibition of histone citrullination and NETs formation	Liver	(18, 190)
			Kidney	(19, 21) (104)
			Cerebral	(115, 119)
			Limb	(128)
			Cutaneous	(129)
	Inhibitors of PAD4 (GSK484, GSK199, nNIF) and PAD4 KO		Liver	(138, 190)
			Kidney	(21, 102, 105)
			Intestinal	(112)
			Lung	(113)
			Cerebral	(115, 117)
			Myocardial	(120)
			Limb	(128)
NADPH oxidase	NADPH oxidase inhibitor: F-apocynin, DPI, TMP	Inhibition of NETs and inflammatory factors	Liver	(91, 97) (99)
	ROS inhibitor: N-acetylcysteine			(90)
MMP9	rAAV8-TIMP-1	Decreased NETs and leukocyte activation	Liver	(92)
HMGB1	HMGB1 antagonist: TM	Inhibition of NETs and NET-induced EMT	Liver	(193)
	Anti-HMGB1 antibody	Decreased NETosis, inflammatory and cell apoptosis	Intestinal	(112)
PD-L1	anti-PD-L1, PD-L1 KO	Decreased NETs	Liver	(192)
***Intracellular signaling molecules***				
MAPK pathway	P38 MAPK inhibitor: Naringin	Inhibition of NETs and inflammatory factors	Liver	(91)
	ERK/JNK inhibitor: TMP			(97)
NF-κB pathway	NF-κB inhibitor: BAY11-7082	Decreased NETs	Kidney	(104)
Syk pathway	Syk inhibitor: R788 disodium			
TLR pathway	Inhibitor of TLR4 (rTM) and TLR4 KO	Inhibition of NETosis and CTC entrapment by NETs	Liver	(99, 191)
			Limb	(126)
	Inhibitor of TLR9 (HCQ) and TLR9 KO	Decreased NETs	Liver	(95)
			Lung	(113)
			Limb	(128)
IL-33/ST2 pathway	IL-33 KO and ST2 KO	Decreased NETs and neutrophil infiltration	Liver	(50)
CCL5-CXCL4	CCL5-CXCL4 blocker: MKEY	Inhibition of NETs	Myocardial	(123)
IL-36R	IL-36R antagonist	Inhibition of NETs	Cutaneous	(129)
P2RX1	P2RX1 inhibitor: NF449	Decreased NETs	Kidney	(105)
PKM2	Inhibitor of PKM2 (ML265) and PKM2 KO	Inhibition of neutrophil activation and NETosis	Cerebral	(118)
Complement cascade	C3 KO	Decreased NETs and neutrophil infiltration	Kidney	(106)
***Interaction of neutrophils with other cells***				
Platelets	anti-CD41 antibody	Inhibition of CTC entrapment by NETs	Liver	(191)
	Clopidogrel	Inhibition of platelet and NETs	Kidney	(101)
	Platelet TLR4 KO	Inhibition of NETs-activated platelets	Liver	(138)
		Inhibition of NETs	Cerebral	(119)
	Platelet HMGB1 KO	Inhibition of NETs	Cerebral	(117)
Mast cells	Depletion of Mast cells	Decreased NETs and neutrophil infiltration	Liver	(93)
***Metabolism-related mechanism***				
Superoxide	Allopurinol	Decreased NETs	Liver	(90)
Gut microbiota	Antibiotic cocktail protocol	Decreased NETs	Myocardial	(125)
Mitochondria	MSC-EVs	Transferred mitochondria and decreased NETs	Liver	(100)
***Others***				
Multiple unknown mechanism	Exercise Training	Decreased NETs and inflammatory network complexity	Liver	(98)
	Drag reducing polymers	Decreased NETs and micrometastases	Liver	(194)
	Histidine-rich glycoprotein	Inhibition of NETs	Liver	(96)
	Tranexamic acid	Decreased NETs	Intestinal	(111)
	Gut microbiota	Inhibition of NETosis	Intestinal	(110)

CTC, circulating tumor cell; DNase I, deoxyribonuclease I; DPI, diphenyleneiodonium; EMT, epithelial to mesenchymal transition; ERK, extracellular regulated protein kinases; HMGB1, high mobility group box 1; IRI, ischemia reperfusion injury; IL, interleukin; MMP9, matrix metallopeptidase 9; MAPK, mitogen-activated protein kinase; MSC-EVs, mesenchymal stromal cell-derived extracellular vesicles; mCBS, methyl β-cellobioside per-O-sulfate; NET, neutrophil extracellular trap; nNIF, neonatal NET-inhibitory factor; NF-κB, nuclear factor kappa-B; PAD, peptidyl arginine deiminase; PD-L1, programmed death ligand 1; P2RX1, purinergic receptor P2X 1; PKM2, pyruvate kinase M2; Refs, references; rTM, recombinant thrombomodulin; ROS, reactive oxygen species; rAAV8-TIMP-1, recombinant adeno-associated virus type 8-tissue inhibitor of metalloproteinases-1; Syk, spleen tyrosine kinase; ST2, suppression of tumorigenicity 2; TMP, tetramethylpyrazine; TLR, Toll-like receptor.

The inhibition of NET formation *via* the targeting of NET components could limit the role of NETs more directly. The blockade of ROS production using antibodies or the inhibition of citrullination histones with PAD4 inhibitors can inhibit NET formation and protect against IRI in the liver (90, 137, 138, 190), kidneys (21, 102, 105), intestine (112), lungs (113), cerebrum (115, 117), myocardium (120), limbs (128), and skin (129). The function of NETs largely depends on their reticular DNA structure and the various proteins embedded within them. Enhancing the clearance of NETs by promoting the degradation of extracellular reticular DNA with DNase I may be another effective strategy to reduce IRI (101, 108). One key advantage of DNase I is that it is used clinically and has not shown any toxicity (195). However, in one study, DNase I treatment could not improve limb IRI, although it promoted NET clearance (127). Therefore, in addition to the reticular DNA structure, the multiple proteins embedded within NETs may also contribute to organ damage. Histones are a core factor causing NET-related tissue injuries, and the positive feedback loop between histones and NETs can aggravate distal organ injury after renal IRI (19). Neutralizing histones on NETs significantly attenuates IRI in multiple organs (19, 109, 124, 162). In addition, the antibody-mediated blockade of other NET components — such as MMP9, HMGB1, and PD-L1 — can also limit the function of NETs and improve IRI (92, 112, 192). NE and MPO are also closely related to NET formation (68, 87). Whether targeting agents against NE and MPO could alleviate organ IRI warrants further investigation.

Some treatments with unknown mechanisms of action, such as exercise training and the supplementation of gut microbiota, can also reduce organ IRI by reducing NETs (98, 110). However, gut microbes have been reported to exacerbate myocardial IRI by regulating NET formation (125). The influence of gut microbiota on organ IRI may be dependent on the species of microorganisms, which leads to the conflicting findings. Immunoregulatory methods for reducing NET formation are currently considered one of the primary therapeutic strategies for organ IRI and may further improve patient outcomes. Future studies are needed to identify therapeutic strategies and drugs that target specific pathways of NET induction, generation, and degradation and evaluate them through clinical trials to demonstrate the potential of NETs as therapeutic targets for organ IRI.

## Current challenges in the study of extracellular traps and ischemia reperfusion injury

Currently, there are some challenges that hinder research on ETs in organ IRI. First, many drugs and treatments that have been reported to inhibit NET formation also inhibit neutrophil infiltration. However, neutrophil is essential for organ repair, thus, blocking of neutrophil infiltration may be deleterious at the late phase of organ IRI (196). In addition, it is worthwhile to investigate whether the decreased neutrophil infiltration causes the decreased NET formation or whether the drug or treatment itself can alter the ability of neutrophils to produce ETs. Second, in organ IRI, many stimuli can trigger cell necrosis, which involves chromatin release similar to NETs. Many reported detection methods cannot distinguish between the generation of extracellular DNA from the release of ETs and the effects of cell necrosis. The combined immunofluorescence staining of DNA and citrullinated histones helps to distinguish between cell necrosis and other forms of DNA release. NE or MPO, the third most important marker, may enhance the reliability of the results if it is found to be co-localized with DNA and histones on staining (23). In addition, the ET release process can be more directly visualized in real-time using intravital confocal microscopy (50). The NET components NE and MPO are also present in METs (26, 197). Therefore, whether the ETs affecting organ IRI at various stages are mainly derived from neutrophils needs to be carefully examined. Comparative analysis using the immunofluorescence-based colocalization of immune cell and ET markers or the depletion of corresponding immune cells can help identify the main sources of ETs (106). Finally, accurate quantification of NETs in patient plasma or serum remains a challenge. Recently, Matta et al. (198) developed a new method to reliably detect NETs in patient plasma using multiplex enzyme linked immunosorbent assay (ELISA) (MPO, citrullinated histone H3 and DNA) combined with immunofluorescence smear methods. The techniques for identifying NETosis are complex. Thus, establishing an ELISA for quantifying NET-related components may be practical. However, the quantitative changes in NET formation do not necessarily translate to disease progression or improvement. Therefore, more studies are needed before ETs can be used as a reliable biomarkers for organ IRI and prognosis.

## Conclusion and prospection

NET formation is a double-edged sword. On the one hand, NETs capture and kill bacteria through their reticular DNA traps, playing an important role in the innate immune defense. On the other hand, the excessive production of NETs can affect the development and outcomes of non-infectious diseases, and especially sterile inflammation-related diseases. NET formation appears to be associated with IRI in various organs, especially in the early stages of inflammatory infiltration following I/R. Recently, the role of METs in liver IRI has also been reported (22). However, the research on ETs at various stages of organ IRI remains in its infancy. In the future, more studies will need to be conducted to explore the roles of ETs generated by various immune cells at different stages of IRI along with the related mechanisms. In addition, it will be necessary to further explore the specific networks regulating ET formation in different microenvironments and the role that the multiple proteins embedded within the reticular DNA traps play in IRI. As numerous proteins present in ETs are investigated, new functions of ETs may emerge. An in-depth understanding of the molecular mechanisms of ET formation could help us inhibit ETs *via* targeted drugs, and then attenuate IRI. Thus, such research could pave the way for new diagnostic and therapeutic strategies for managing IRI.

## Author contributions

FZ and YL wrote the manuscript. FZ, YL and JW contributed to conception, design and interpretation of the manuscript. JZ, PC and ZS revised the manuscript. WW supervised the manuscript. All authors contributed to the article and approved the submitted version

## Acknowledgments

We thank that this work was supported by the National Natural Science Foundation of China (NSFC, 81771720 and 82070764) and the Natural Science Foundation of Beijing (7212040). Thanks to all of our team for their help with the manuscript.

## Conflict of interest

The authors declare that the research was conducted in the absence of any commercial or financial relationships that could be construed as a potential conflict of interest.

## Publisher’s note

All claims expressed in this article are solely those of the authors and do not necessarily represent those of their affiliated organizations, or those of the publisher, the editors and the reviewers. Any product that may be evaluated in this article, or claim that may be made by its manufacturer, is not guaranteed or endorsed by the publisher.
